# Calcium interacts with temperature to influence *Daphnia* movement rates

**DOI:** 10.1098/rsos.160537

**Published:** 2016-12-07

**Authors:** Gustavo S. Betini, Jordan Roszell, Andreas Heyland, John M. Fryxell

**Affiliations:** Department of Integrative Biology, University of Guelph, Guelph, Ontario, CanadaN1G 2W1

**Keywords:** aquatic systems, climate change, *Daphnia magna*, diffusion, turn rates, velocity, zooplankton

## Abstract

Predicting the ecological responses to climate change is particularly challenging, because organisms might be affected simultaneously by the synergistic effects of multiple environmental stressors. Global warming is often accompanied by declining calcium concentration in many freshwater ecosystems. Although there is growing evidence that these changes in water chemistry and thermal conditions can influence ecosystem dynamics, little information is currently available about how these synergistic environmental stressors could influence the behaviour of aquatic organisms. Here, we tested whether the combined effects of calcium and temperature affect movement parameters (average speed, mean turning frequency and mean-squared displacement) of the planktonic *Daphnia magna*, using a full factorial design and exposing *Daphnia* individuals to a range of realistic levels of temperature and calcium concentration. We found that movement increased with both temperature and calcium concentration, but temperature effects became considerably weaker when individuals were exposed to calcium levels close to survival limits documented for several *Daphnia* species, signalling a strong interaction effect. These results support the notion that changes in water chemistry might have as strong an effect as projected changes in temperature on movement rates of *Daphnia*, suggesting that even sublethal levels of calcium decline could have a considerable impact on the dynamics of freshwater ecosystems.

## 1. Background

The response of organisms to climate change depends on the individual effect of many environmental stressors, but also on the way they potentially interact with each other to affect survival and reproduction [1–4]. Changes in temperature, nutrients and the increase of extreme events can interact to generate nonlinear effects that are difficult to predict with single-stressor studies [3]. For example, long-term changes in planktonic consumer and producer biomass are best explained by the interaction among warming, drought and acidification, rather than among the sum of their individual effects [3].

Recent changes in water chemistry in both marine and freshwater systems are triggering a number of ecological impacts on primary producers, creating cascade effects across entire aquatic food webs [5–7]. A major challenge for aquatic organisms is the reduced levels of available calcium (Ca) concentrations in soft water boreal lakes such as in eastern North America and western Europe [8–10]. This reduction is part of a natural and long-term process of soil acidification, but it has been accelerated by anthropogenic activities that influence calcium cycling, such the forest harvesting and the elevated rates of Ca leaching over several decades of highly acidic precipitation. In the case of harvest, removal of timber followed by several cycles of regrowth of forests results in a decline of Ca in soils and, consequently, a decline in the amount of Ca that reaches the lakes via run-off process [11]. Because Ca is an essential element for cellular function and structural support that allows many zooplankton species to grow in size, Ca decline can influence growth, survival and development of many freshwater organisms [11–13]. *Daphnia* species have been particularly affected because of their high demand for Ca during their frequent moults during both juvenile and adult stages, although this could vary greatly with species and even among different populations [8,9,13]. Nevertheless, *Daphnia* populations in software lakes, which are naturally low in Ca, have been replaced by Ca-poor species such as the crustacean *Holopedium glacialis* [7]. An increase in the *Holopedium* population could reduce energy and nutrient transport in lake food webs. This could happen, because *Holopedium* has lower P and Ca content than *Daphnia,* the dominant and keystone herbivores in pelagic zone in both Europe and North America. As planktivorous fish feed more on poor content Ca and P organisms, fewer essential nutrients would be available at higher trophic levels [7].

Despite the well-known effects on survival and reproduction, recent changes in dissolved Ca in freshwater systems could also affect the behaviour of aquatic organisms. For example, an extended period of development caused by low Ca intake could create trade-offs between growth and swimming or feeding, which could ultimately affect fitness. For aquatic organisms such as *Daphnia,* movement is particularly important because it allows individuals to track resource abundance and optimal environmental conditions for reproduction and growth [10,14–16]. Swimming in filter feeders is also positively related to feeding [17,18] and changes in swimming speed would accordingly have a direct effect on reproduction and survival. Moreover, Ca and temperature have been shown to interact to affect growth, reproduction and development in *Daphnia* species [12,13,19]. Given that movement is directly related to temperature via increase in metabolic rate [20], and that variation in temperature caused by recent climate change is perhaps the most widely recognized environmental stressor influencing ecological processes worldwide [21–24]*,* it is likely that potential effects of Ca on movement of individuals in natural populations might be influenced by variation in both Ca and mean temperature. Temperature can vary widely among lakes and within the water column, and climate change is expected to increase these differences [25]. For example, in northern and southcentral Ontario, Canada, where decline in Ca has been observed in many lakes, climate change is expected to result in an increase in surface water temperature up to 5°C [25]. Although many studies have addressed the ecological consequences of global warming on behaviour [26–28] and the recent changes in Ca as an environmental stressor [7,8,11,29], there is no information available on whether temperature and Ca interact to affect the movement of aquatic organisms.

Here, we investigate the combined effects of Ca and temperature on movement parameters of the water flea *Daphnia magna*. We used a balanced factorial design, rearing adults in 16 different combinations of Ca concentration and temperature that are usually found in natural lakes. We then measured movement in their offspring and calculated three key parameters influencing mobility: mean-squared displacement, average speed and turn frequency. This experimental design allowed us to investigate both the isolated and combined effects of calcium and temperature on the movement behaviour of *Daphnia*.

## 2. Methods

### 2.1. Movement assays

The clonal population of *Daphnia* used in the movement experiment were reared in COMBO-ANIMATE medium [30] kept in 20°C, 24 h light and fed with the algae *Chlorella vulgaris* that were kept in the same medium and under the same temperature and light conditions. Ca concentration in this medium was 10 mg l^−1^. To investigate the effects of both temperature and Ca on the movement of *Daphnia*, we used a full factorial design where individuals were reared in 16 treatment combinations: four different temperatures (16°C, 20°C, 24°C and 28°C) and four different Ca concentrations (1, 11, 21 and 31 mg l^−1^). Both the temperature and Ca concentrations used are commonly found in boreal lakes [25,31]. Ca levels around 1.5 mg l^−1^ are the limit of reproduction and survival recorded for *Daphnia*. To adjust the Ca concentration, we used the same COMBO medium, but changed the amount of CaCl_2_ H_2_O used in the regular medium (i.e. Ca = 10 mg l^−1^). Each treatment was initiated with 10 individuals in 250 ml flasks with 230 ml of adjusted COMBO to meet the Ca concentration of a given treatment and 20 ml of *C. vulgaris* reared in adjusted COMBO and constant temperature (20°C). Every 3 days, approximately 230 ml of the medium was changed via reverse filtration and 20 ml of the same *C. vulgaris* was added.

We measured the movement of 10 individuals born in each treatment combination that were between 3 days old and development of first clutch (day 0 = the day they were released into the water by their mother). We used juveniles, because the Ca requirement of juveniles is typically higher than that of adults, probably owing to the increased frequency of moulting during juvenile growth [8,13]. Trials with the same combination of Ca and temperature were never conducted at the same time of the day or in the same order.

To simplify movement behaviour in a one-dimensional space and access mobility indexes traditionally used to quantify animal movement, we measure all individual *Daphnia* using a glass pipette of 30 × 0.8 cm. They were first removed from each of the 16 different stock cultures where they were reared and placed in 50 ml of adjusted COMBO solution at room temperature for at least 5 min before the beginning of each trial to allow habituation to the new environment and avoid variation in swimming behaviour caused by temperature [32]. They were then placed in the glass pipette with 20 ml of that medium. The pipette was immediately capped at both ends, and each individual was left for 5 more minutes inside the pipette before the beginning of each trial. During each 5 min trial, turn times, distances between turns and directions were recorded using a ruler placed alongside the pipette. We defined a step as the distance travelled by each individual between turns, with turns being operationally defined as reversal in movement direction at 1 cm resolution [33,34].

### 2.2. Mobility indexes

Because of the size of the glass pipette, individuals were able to move only in one dimension, meaning that each individual could control their displacement by changes in their speed and/or turn frequency. To characterize the movement of *Daphnia,* we used three different indices of mobility: average speed, mean turning frequency and mean-squared displacement of each individual, in each treatment combination. Speed was calculated as the distance travelled between turns divided by the duration of the movement step (in cm per second). Mean turning frequency was calculated as the total number of turns divided by 5 min (the length of each trial), averaged for each individual. Mean-squared displacement was the square of the distance travelled between turns, averaged by each individual [33].

### 2.3. Statistical analysis

We used a generalized least-squares regression (GLS) to investigate whether calcium and temperature interacted to affect the movement behaviour of *Daphnia.* Using Akaike information criteria (AIC) [35], we compared four different models: two models with or without the interaction term as explanatory variable and two other models with the single effects of Ca or temperature. We used a variance structure to control for heterocedasticity that was caused by an increase in the spread of the residuals with increasing Ca concentration. The variance structure had the following form: var (ϵ*_i_*) = *σ*^2^ × |Ca_*i*_|^2*δ*^, where ϵ*_i_* are the residuals, *σ*^2^ is the variance of the residual, Ca*_i_* are the four Ca concentrations used in the experiment and *δ* is a constant to be estimated from the data [36]. All analysis was performed in R [37]. We used the package *nlme* to perform the GLS [38] and the package *visreg* to create the conditional plots [39].

## 3. Results

**Figure 1.**
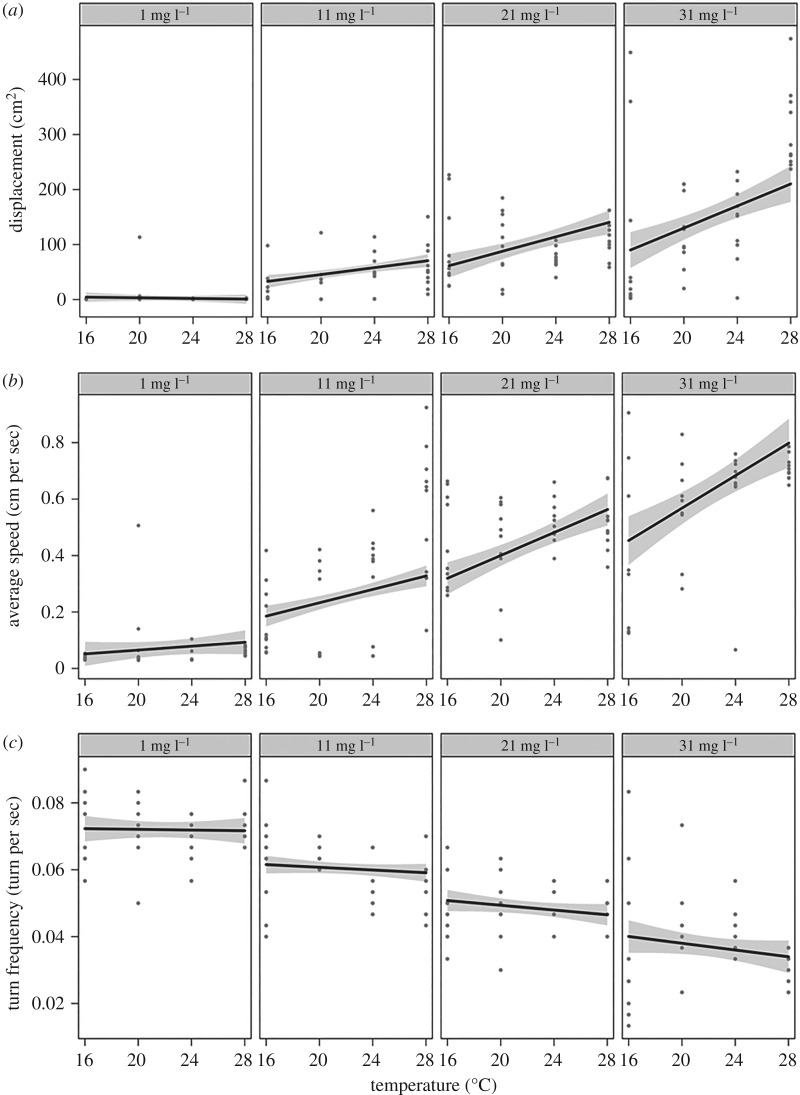
Conditional plots illustrate the effect of calcium and temperature on the movement of *D. magna*. Conditional plots were calculated from the models with (*a*) mean-squared displacement, (*b*) average speed or (*c*) mean turning frequency as response variables, and the interaction between temperature and calcium as explanatory variable. According to Akaike's information criterion, variation in mean turning frequency was equally explained by the model with the interaction and the model with only calcium as explanatory variable. Dots represent partial residuals, black lines are prediction lines and grey-shaded areas are confidence intervals based onthe model.

We found that a model with an interaction between temperature and Ca was the most parsimonious in explaining variation in mean-squared displacement (tables 1 and 2 and figure 1*a*); mean-squared displacement increased with temperature but only at higher levels of Ca concentration (figure 1*a*). The model with the interaction term also offered the most parsimonious explanation for variation in average speed (tables 1 and 2 and figure 1*b*); average speed increased with temperature, but not for the lowest level of Ca (figure 1*b*). Variation in mean turning frequency was best explained by a model with only Ca as explanatory variable (tables 1 and 2 and figure 1*c*), although, based on AIC, there was no evidence to dismiss the model with Ca and temperature and the model with their interaction as potential candidates (table 1).

**Table 1.** Akaike's information criterion (AIC) parameters for competing models used to explain variation in movement behaviour of *D. magna* exposed to different calcium and temperature treatments. d.f., degrees of freedom; LogLik, log-likelihood value of each model; AICc, AIC corrected for small samples; ΔAICc, difference for model relative to the smallest AICc in the model set; *w*, Akaike weight, which is the approximate probability in favour of the given model from the set of models considered.

model	d.f.	LogLik	AICc	ΔAIC	*w*
mean-squared displacement					
1. Calcium × temperature	6	−846.003	1704.6	0.00	0.999
2. Calcium + temperature	5	−854.964	1720.3	15.76	0.000
3. Calcium	4	−855.377	1719.0	14.46	0.001
4. Temperature	4	−907.874	1824.0	119.45	0.000
average speed					
1. Calcium × temperature	6	72.331	−132.1	0.00	0.994
2. Calcium + temperature	5	66.057	−121.7	10.39	0.006
3. Calcium	4	59.334	−110.4	21.70	0.000
4. Temperature	4	−2.348	13.0	145.07	0.000
mean turning frequency					
1. Calcium × temperature	6	507.796	−1003.0	1.59	0.198
2. Calcium + temperature	5	507.327	−1004.3	0.37	0.365
3. Calcium	4	506.444	−1004.6	0.00	0.438
4. Temperature	4	430.718	−853.2	151.45	0.000

**Table 2.** Parameter estimate, lower and upper values of the 95% confidence intervals for the most parsimonious model used to explain movement behaviour in *D. magna*. Models were selected using AIC model competition.

model	lower	estimate	upper
mean-squared displacement			
1. Intercept	−15.10	11.90	38.91
2. Ca × temperature	0.18	0.34	0.50
3. Calcium	−6.15	−2.65	0.85
4. Temperature	−1.84	−0.64	0.56
speed			
1. Intercept	−1.37 × 10^−1^	−2.69 × 10^−3^	1.32 × 10^−1^
2. Ca × temperature	3.82 × 10^−4^	8.45 × 10^−4^	1.31 × 10^−3^
3. Calcium	−1.05 × 10^−2^	−1.478 × 10^−4^	1.02 × 10^−2^
4. Temperature	−3.44 × 10^−3^	2.57 × 10^−3^	8.57 × 10^−3^
mean turning frequency			
1. Intercept	7.07 × 10^−2^	7.31 × 10^−2^	7.55 × 10^−2^
3. Calcium	−1.30 × 10^−3^	−1.16 × 10^−3^	−1.03 × 10^−3^

## 4. Discussion

Here, we provide empirical evidence that the interaction between temperature and Ca concentration played a key role influencing variation in movement rates of the aquatic invertebrate *D. magna*. We showed that mean-squared displacement increased with both Ca concentration and temperature, but only for Ca levels well above the critical limits for reproduction and survival recorded for *Daphnia* (greater than 1.5 mg l^−1^; [8,29]). Individuals exposed to high concentrations of Ca had also high average speed and low turn frequency, i.e. they moved longer distances into the glass pipette before turning compared with individuals exposed to low levels of Ca or high temperature.

A number of empirical studies have demonstrated that the combined effects of different stressors sometimes offers a better explanation of organismal response to climate change than single factors in isolation [1,3,4]. For example, interaction effects were found in 74% out of 171 studies encompassing individuals, populations and whole communities in marine ecosystems [40]. Because the list of chemical, physical and biological stressors potentially dangerous to the environment has grown rapidly [1,3], the need for multifactorial research designs is becoming increasingly obvious to understand how stressors can potentially affect aquatic invertebrates. Such multifactorial designs are certainly more time consuming than simpler single-factor experiments, which might be the reason why they are rare in the ecological literature [40]. However, we submit that only by examining the combined impact of multiple stressors will we gain a robust understanding of the potential impact of human-induced (i.e. climate change) and natural variation of temperature on organisms, populations and ecosystems [40].

Our results suggest that single effects of temperature might not be the only important consequence of climate change for aquatic invertebrates. We show that a model to explain variation in the movement behaviour of *Daphnia* with only temperature as explanatory variable was poorly supported by our data. Temperature had no detectable effect on movement at the lowest Ca treatment, a concentration that is increasingly common in many natural lakes (e.g. 35% of 770 lakes in southeastern Canada had Ca concentrations below 1.5 mg l^−1^ and 62% of 1200 lakes in Norway had median values around 1 mg l^−1^) [6,11]. Although empirical studies have documented that temperature can affect several key aspects of the ecology of *Daphnia* species [26,28,41,42], previous studies have been conducted with temperature as the single environmental stressor. Our own results and recent empirical evidence suggest that the negative effects of climate change on organisms act via interactions among different stressors [40]. Thus, it is possible that changes in water chemistry acting in synergy with ongoing increasing trends in temperature might have a more widespread and pronounced impact on aquatic ecosystems than is widely appreciated[6].

At this point, the mechanistic basis for changes in movement of *Daphnia* in response to changing Ca concentrations is unclear. It could be the case that development is compromised when individuals are exposed to low levels of Ca, leading to small body size [11] and, consequently, reduced swimming ability [32]. In addition, variation in Ca uptake capacity has been linked with changes in locomotor activity in crustaceans [43] and it is therefore reasonable to assume such a link in *Daphnia*. More importantly, new insights into the mechanisms of Ca uptake in *Daphnia* [13,44,45] might shed further light on the interaction between Ca and temperature. For example, low temperatures may increase intracellular calcium concentrations by preventing calcium from exiting the cell, further affecting cell excitability. In muscle cells, high intracellular [Ca^2+^] can decrease adenosine triphosphate availability. Both responses can lead to reduced muscle contractions and consequently reduced locomotor ability.

So far, studies trying to understand the ecological consequences of low Ca in natural lakes have focused on the effects of Ca on fecundity and survival [7,11,13]. According to our study, low Ca could also negatively impact the behaviour of aquatic invertebrates, which is probably owing to the negative impacts of low Ca on development (e.g. small body size and/or Ca uptake capacity). *Daphnia* species perform daily movement into the water column to avoid predation and increase feeding, growth and reproduction [15,16,46,47]. Thus, it is also likely that the recent decline in abundance of Ca-rich species could be in part explained by the inability of individuals to track resources or escape from predators when exposed to low Ca, which has been experimentally demonstrated [48].

Aquatic ecosystems worldwide are experiencing an increase in water temperature, which is expected to alter ecological processes of natural populations, in addition to the well-described changes in distribution, seasonal phenology and morphology of many species [22,27,28,49–51]. Ca limitation is now a recognized threat because of documented decline in soft water lakes in both North America and Europe [6,52]. As a consequence, *Daphnia* species, the keystone herbivores in pelagic food webs, have been nearly extirpated in many lakes in North America [6]. Here, we show that, under laboratory conditions, these two important environmental stressors interact to affect movement behaviour of *D. magna*.

Although the levels of both temperature and Ca used in our experiment are typical of many boreal lakes [6,11,52], we believe it will be important in future studies to investigate how clones from natural lakes respond to these stressors. Individuals from natural populations might be better adapted to low Ca and changes in temperature than laboratory populations [11]. Whether these adaptations are plastic or genetic responses to environmental conditions, they both could influence the response of *Daphnia* to stressful situations. In addition, Ca could also interact with other elements to affect the behaviour of aquatic invertebrates. For example, many low-calcium lakes are also contaminated with copper, which has been shown to be less toxic at low Ca concentrations [45] and phosphorus, another nutrient that has been in decline in boreal lakes, can also interact with Ca to affect individual growth and survival [53]. Nevertheless, our study is the first, to the best of our knowledge, to show that the synergistic effects of temperature and Ca better explain variation in movement rates by aquatic invertebrates.

## Data accessibility

The data available represent movement data for each individual and treatment level used in the experiments (position relative to the ruler, time and distance of each step). Data available from the Dryad Digital Repository [54]: http://dx.doi.org/10.5061/dryad.v09hh.
